# MOnitored supplementation of VItamin D in preterm infants (MOSVID trial): study protocol for a randomised controlled trial

**DOI:** 10.1186/s13063-017-2141-y

**Published:** 2017-09-11

**Authors:** Alicja Kołodziejczyk, Maria K. Borszewska-Kornacka, Joanna Seliga-Siwecka

**Affiliations:** Neonatal and Intensive Care Department, Medical University of Warsaw, Karowa 2 Street, 00-315 Warsaw, Poland

**Keywords:** Vitamin D, Osteopenia, Prematurity

## Abstract

**Background:**

The pivotal role of vitamin D (vit D) in skeletal health is well known. Neonatal vit D storage at birth is dependent on maternal levels, and newborns receive 50–70% of their mother’s 25-hydroxyvitamin D [25(OH)D]. Deficiency of vit D can lead to prematurity bone disease, with an incidence of up to 55% in infants weighing < 1000 g. The aim of this study is to assess the effectiveness of monitored supplementation of vit D in a population of preterm infants.

**Methods/design:**

Preterm infants born at 24–32 weeks of gestation will be recruited within the first 7 days of life. Depending on the type of feeding, and after reaching partial enteral feeding or at 7 days of life, vit D supplementation will consist of 500 IU and an additional 150–300 IU/kg included in human milk fortifiers (if fed exclusively with breast milk) or 190 IU/kg in milk formulas. Subjects will be randomised to either monitored (with an option of dose modification based on 25(OH)D levels as per protocol) or standard therapy up to 52 weeks of post-conceptional age (PCA). The primary outcome measure will be the number of neonates with deficiency or excess levels of 25(OH)D at 40  ±2 weeks of PCA. Additional 25(OH)D levels will be measured at birth, at 4 and 8 weeks of age, and/or at 35 and 52  ±2 weeks of PCA. Secondary objectives will include the incidence of osteopenia, nephrocalcinosis and nephrolithiasis. Serum parameters of calcium phosphorus metabolism will also be measured.

**Discussion:**

Despite multiple years of research and numerous publications, there is still a lack of consensus in regard to *how much* vit D infants should receive and *how long* they should receive it. Because 80% of calcium and phosphorus placental transfer occurs between 24 and 40 weeks of gestation, preterm infants are especially prone to adverse effects of vit D insufficiency. However, both inadequate and excessive amounts of vit D may be unsafe and lead to serious health issues. The results of our study may shed new light on these concerns and contribute to optimising vit D supplementation.

**Trial registration:**

ClinicalTrials.gov, NCT03087149. Registered on 15 March 2017.

**Electronic supplementary material:**

The online version of this article (doi:10.1186/s13063-017-2141-y) contains supplementary material, which is available to authorized users.

## Background

The pivotal role of vitamin D (vit D) in skeletal health is well known. The discovery of receptors for vit D in most tissues and cells has provided new insights on its role in non-skeletal health. These actions include, among others, regulation of cellular proliferation, apoptosis and angiogenesis [1].

Studies have shown that in both term and preterm infants, neonatal vit D storage at birth is dependent on maternal 25-hydroxyvitamin D [25(OH)D] because the foetus secures all its vit D from the mother. At birth, regardless of gestational age (GA), neonatal 25(OH)D levels are 50–70% derived from mother’s [2–4].

In the population of infants born prematurely, vitamin D deficiency (VDD) can lead to prematurity bone disease, which is described by different names as rickets of prematurity, osteopenia of prematurity or metabolic bone disease (MBD) of prematurity. The incidence of this morbidity can reach up to 55% in infants weighing < 1000 g [5]. In this group of neonates, VDD may also lead to an increased risk of respiratory tract infection and chronic respiratory morbidity such as bronchopulmonary dysplasia (BPD), as well as seizures and growth disturbance [6–10].

In order to prevent VDD, recommendations for vit D supplementation have been published. The American Academy of Pediatrics guidelines recommend supplementation of 200–400 IU/day in enterally fed preterm infants [11], whereas in Europe, according to European Society for Paediatric Gastroenterology, Hepatology and Nutrition (ESPGHAN) guidelines, supplementation of vit D for preterm infants should reach 800–1000 IU/day [12].

Recently, separate guidelines have been published for Central Europe. Preterm infants fed enterally should receive vit D supplementation of 400–800 IU/day within the first days of life and continue up to 40 weeks of GA. This should be followed by 400 IU/day [13].

Researchers in two prospective observational studies have assessed recent recommendations for vit D supplementation. Monangi et al. [14] published a paper evaluating vit D status in 120 preterm infants (≤32 weeks of gestation) at birth and at 36 weeks post-menstrual age or discharge. Daily vit D intake was based on parenteral nutrition, human milk, human milk fortifier or milk formula, as well as vit D supplementation of 200 IU/day. Proportions of 64% of infants at birth and 35% infants at 36 weeks of post-conceptional age (PCA) or at discharge had serum 25(OH)D concentrations < 20 ng/ml. The authors concluded that low vit D status at birth and suboptimal vit D intake were responsible for insufficient 25(OH)D levels in the population of studied infants [14].

A study conducted by Pinto et al. [15] in Australia in 2015 evaluated vit D status in 28 infants (30–36 weeks of gestation) at birth and at 36 weeks PCA [15]. Compared with Monangi et al.’s trial, total daily vit D was increased due to higher vit D supplementation of 400 IU/day. The proportion of infants with VDD decreased from 32.1% to 7.1%, (*p* = 0.016). However, this study was small in numbers and excluded extremely low birth weight (ELBW) infants, in whom the risk of VDD is the highest [15].

Two randomised studies have become available since the publication of new recommendations for vit D supplementation among preterm infants. In a randomised, double-blind trial done in North India, investigators enrolled 96 infants (28–32 weeks of GA) in two groups: vit D 400 IU/day vs vit D 800 IU/day. The primary outcome was VDD at 40 weeks of post-menstrual age. Secondary outcomes included VDD, bone mineral content and density at 3 months of corrected age (CA). The prevalence of VDD (defined as < 20 ng/ml) in the 800 IU/day group was significantly lower than in the 400 IU/day group at 40 weeks of PCA (38.1% vs 66.7%, relative risk [RR] 0.57, 95% CI 0.37–0.88) and at 3 months of CA (12.5% vs 35%, RR 0.36, 95% CI 0.14–0.90). One infant in the 800 IU/day group had vit D hypervitaminosis (> 100 ng/ml). Bone mineral content and bone mineral density did not differ between groups. Again, this trial did not included infants born ELBW, who are at most risk of VDD, as well as supplementation-induced hypervitaminosis, immediately after birth. Considering these factors, we hypothesise that this population would probably benefit the most from sufficient vit D supplementation [16].

Researchers in a U.K. trial randomised 100 infants (from 23 to 28 weeks of GA) to three groups: placebo (routine vit D supplementation in parenteral and enteral nutrition), 200 IU (additional 200 IU/day) or 800 IU (additional 800 IU/day). Infants in the 800 IU/day group presented with higher 25(OH)D concentration (*p* < 0.05). The incidence of death, BPD, necrotizing enterocolitis (NEC) or intracranial haemorrhage did not differ between the study groups. The authors concluded that ESPGHAN recommendations led to overdosing vit D (> 50 ng/ml) in many infants, whereas routine intake of an additional 200 IU/day allowed more infants to reach recommended levels [17].

As shown by the above studies, inappropriate vit D supplementation may lead to VDD or vitamin overdosing and mild hypercalcemia [18]. The dosage, safety and effectiveness of vit D supplementation in preterm infants remain controversial topics. Clear criteria for adequate 25(OH)D levels in preterm infants have not been established. In view of inconsistent and insufficient data, several authors have suggested that vit D supplementation should be monitored in the preterm population [10, 11].

The main objective of the present trial is to assess the effectiveness of monitored supplementation of vit D in a population of preterm infants born at 24–32 weeks of gestation with the aim of optimising 25(OH)D levels measured at 4 and 8 weeks of age and/or 35, 40 and 52 weeks of PCA. We hypothesise that monitored therapy is more effective and safer than standard therapy in infants given vit D supplementation. Secondary objectives are to assess the effect of vit D on the prevalence of osteopenia, nephrocalcinosis and nephrolithiasis.

## Methods/design

### Study design

We are conducting a pragmatic, unblinded, parallel-group, randomised controlled superiority trial.

### Setting and participants

Infants born at 24–32 weeks of gestation will be considered for inclusion. Parents will be approached shortly after birth and admission to the neonatal intensive care unit at Princess Anne Hospital in Warsaw, Poland (a tertiary level perinatal centre, Neonatal and Intesive Care Department, Medical University of Warsaw). Caregivers of eligible infants will be invited to take part in the study. After providing oral and written information about the study, informed consent will be obtained by one of the research team members. Preterm infants will be randomly assigned to a monitored group or a standard group. Later, a blood sample will be obtained within the first week of life to assess 25(OH)D levels at birth. At day 7 of life or when reaching 100 ml/day of enteral feeding, all infants will receive 500 IU of vit D with an additional 160 IU/kg of vit D included in parenteral nutrition. Infants will receive supplementation up to 52 weeks of PCA. All procedures will take place at the Neonatal and Intensive Care Department, Medical University of Warsaw.

### Inclusion criteria

We will include all preterm infants born between 24 and 32 weeks of gestation (outborns must be admitted within 48 h after delivery). At the time of recruitment, caregivers must be willing to return for follow-up visits and provide written informed consent.

### Exclusion criteria

We will exclude the following infants: those born at > 32 weeks of gestation, and those with major congenital abnormalities, cholestasis or severe illness at birth deemed incompatible with survival. Exclusion criteria will also include lack of written informed consent as well as communication difficulties with caregivers.

### Randomisation criteria

Participants will be randomised within the first 7 days of life after re-evaluating inclusion and exclusion criteria.

### Interventions

**Fig. 1:**
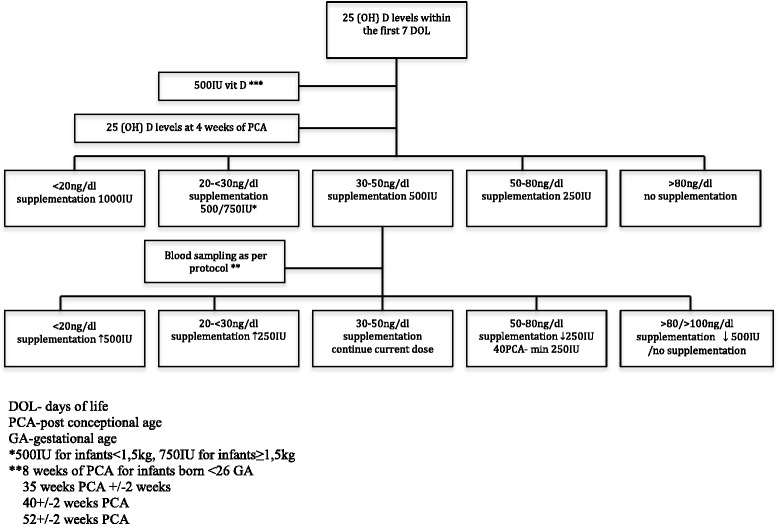
Treatment allocations *500 IU for infants weighing < 1.5 kg, 750 IU for infants ≥ 1.5 kg. **8 weeks of PCA for infants born at < 26 of GA, *** vit D from DOL 7, ﻿35 ± 2 weeks of PCA, 40 ± 2 weeks of PCA, 52 ± 2 weeks of PCA. *DOL* Days of life, *PCA* Postconceptional age

Initially, all infants will receive 500 IU of vit D (cholecalciferol/Devikap; Polpharma, Starogard Gdański, Poland). After full enteral feeding is reached, depending on the type of feeding, vit D supplementation will consist of 500 IU and 150–300 IU/kg included in human milk fortifiers (if fed exclusively with breast milk) or 190 IU/kg in milk formulas. At 4 weeks of age, blood samples for 25(OH)D levels will be obtained, followed by subsequent measurements at 8 weeks of age and/or 35, 40 and 52 weeks of PCA. In the monitored group, vit D doses will be appropriately modified on the basis of 25(OH)D levels (Fig. 1). We hypothesise that most cases of VDD in infants will be secondary to an initial low maternal vit D level. Thus, in these cases, we are planning to increase vit D supplementation by 500 IU because this will allow reaching a higher recommended level of 1000 IU/day [12].

Infants randomised to standard therapy will only have blood samples obtained in the same fashion as the monitored group, but no dosing changes will be applied.

### Allocation concealment and blinding

Opaque, sealed envelopes labelled with consecutive study numbers will be allocated to included patients. The block size will be blinded until completion of the study. These envelopes will contain index cards with the allocated treatment. The statistical team, which will not take part in enrolling infants or in follow-up, will generate the allocation sequence. The randomisation list will remain with the statistical team for the duration of the study. Neonates fulfilling inclusion criteria will be enrolled after admission. A member of the recruitment team will randomise enrolled patients. He/she will open an envelope labelled with the allocated participant number and inform the primary care physician about the study group. Due to the nature of the intervention, neither participants nor physicians applying the intervention can be blinded to allocation. A staff member not involved in recruitment and treatment of patients will collect appropriate data to allow researchers to analyse results without having access to information about the allocation. All patient and study information will be stored on a secure, password-protected, Web-based platform.

#### Compliance

Prior study initiation, an introductory meeting will be scheduled. The session will include the following:

- Brief presentation of the study
- Instructions about randomisation for personnel who will take part in patient allocation
- Instructions about monitored supplementation and standard supplementation

A subsequent meeting will take place 2 months after the start of the study. Staff will be asked about any problems they might be experiencing with implementing the study, such as patient recruitment, randomisation and treatment allocation. Prior to discharge, caregivers of included infants will receive oral and written instructions about vit D application. Depending on the neonate’s PCA, follow-up visits will be scheduled as per protocol.

### Primary outcome

The number of neonates with deficient or excess 25(OH)D levels at 40 ± 2 weeks of PCA will be the primary endpoint. Additional 25(OH)D levels will be measured at birth; at 4 and 8 weeks of age; and/or at 35 and 52 ± 2 weeks of PCA. Venous blood samples will be collected into glass specimens by neonatal nurses to assess 25(OH)D levels in pre-specified time frames (Fig. 1). An automated quantitative test available at the study site, VIDAS® (bioMérieux, Marcy l’Etoile, France), will be used to measure 25(OH)D. The analyser’s calibration will be checked with appropriate controls as per product guidelines. The definitions for vit D status differ slightly between local and global health authorities [13, 19, 20]. On the basis of our geographical location and the fact that none of the published documents offer a separate 25(OH)D reference range for preterm infants, we chose to follow the most recent recommendations for Central Europe [13]:

- Deficiency: 0–20 ng/ml (0–50 nmol/L)
- Suboptimal concentration: > 20–30 ng/ml (> 50–75 nmol/L)
- Optimal concentration: > 30–50 ng/ml (> 75–125 nmol/L)
- Increased level: > 50–100 ng/ml (125–250 nmol/L)
- Potentially toxic level: 100–200 ng/ml (250–500 nmol/L)
- Toxic level: > 200 ng/ml (> 500 nmol/L)

### Secondary outcomes

#### Osteopenia

The exact incidence of osteopenia remains unknown, in part owing to the lack of consensus on its definition. We have chosen to define MBD as decreased bone mineral content relative to the expected level of mineralisation for a foetus or infant of comparable size or GA, seen in conjunction with biochemical and/or ultrasound changes. Neonatal nurses will collect venous samples for serum alkaline phosphatase (ALP) and phosphate (P) levels at 35, 40 and 52 ± 2 weeks of PCA. The AU480 chemistry analyser (Beckman Coulter, Brea, CA, USA) will be used to perform the measurements. The analyser’s calibration will be checked with appropriate controls as per product guidelines.

Additionally, we plan to assess average bone mass (ABM) using quantitative ultrasound (Sunlight PREMIER 7000; BeamMed, Petah Tikva, Israel). This safe, non-invasive, radiation-free and easy-to-use method has been suggested as a screening tool for detecting osteopenia in premature infants [21–23]. With placement of a small ultrasound probe (CRB probe RoHS 900–1000 kHz) along the mid-tibia, this device measures speed of sound (SOS) in meters per second in the axial transmission mode. High intra-individual variation does not allow definition of normal values. However, in a recently published study, preterm infants (24–28 weeks of GA) examined at 40 weeks of PCA showed significantly lower SOS than term infants [22]. In order to evaluate infants receiving monitored vit D therapy, SOS will be higher than with standard therapy, indicating increased ABM. Two previously trained neonatologists not participating the study and blinded to group allocation will assess ABM in each enrolled patient at 35 and at 40 ± 2 weeks of PCA. The measurements will be made on the tibia. The mid-tibial shaft length will be determined by measuring the distance from the knee to the heel. The probe will be placed over the medial aspect of the mid-shaft tibia to obtain an SOS measurement. Three measurements will be performed. The mean value of these measurements will be used for the data analysis. We decided to define MBD as serum levels of ALP > 500 IU and P < 1.8 mmol/L [24].

#### Nephrocalcinosis and nephrolithiasis

Neonatal nurses will collect venous samples for serum and urine calcium, P and creatinine levels at 35, 40 and 52 ± 2 weeks of. The AU480 chemistry analyser will be used to perform the measurements. The analyser’s calibration will be checked with appropriate controls as per product guidelines. Hypercalcaemia will be defined as serum levels ≥ 2.65 mmol/L. Hypercalciuria will be measured by calculating urine calcium/creatinine ratios [25–27]. Both urine calcium and creatinine are risk factors for nephrolithiasis in infants. P deficiency suppresses parathyroid hormone activity and initiates 1,25-(OH)_2_D synthesis, leading to hypercalcaemia, hypercalciuria and increased P kidney reabsorption. Tubular reabsorption of phosphate (TRP) is a widely accepted indicator of inadequate P intake. TRP is calculated from phosphorus/creatinine ratio in the urine and serum. TRP > 95% with P < 1.8 mmol/L is highly suggestive of osteopenia [28].

In preterm infants, ultrasonography has proven good intra-observer reproducibility (kappa = 0.84) and is a reliable tool for detecting nephrocalcinosis [29]. A trained ultrasonographer will assess subjects for nephrolithiasis at 35 and 52 ± 2 weeks of PCA using the HD11 XE ultrasound system (Philips Healthcare, Andover, MA, USA). Increased medullar echogenicity (small white flecks in the tip of the pyramids) will be considered as nephrocalcinosis [30]. Photographic documentation will be obtained.

#### Adverse events

We will define an adverse event as any untoward medical occurrence in a subject without regard to the possibility of a causal relationship. Adverse events will be collected after the subject has provided consent and is enrolled in the study. All adverse events occurring after entry into the study and until hospital discharge will be recorded. An adverse event that meets the criteria for a serious adverse event (SAE) between study enrolment and hospital discharge will be reported to the local ethics committee. An SAE for this study is any untoward medical occurrence that is believed by the investigators to be causally related to the study intervention and results in any of the following: life-threatening condition (that is, immediate risk of death), severe or permanent disability, and prolonged hospitalisation. SAEs occurring after a subject is discontinued from the study will not be reported, unless the investigators feel that the event may have been caused by the study drug or a protocol procedure.

### Retention of participants in the study

Because most of the included patients face long-term hospital care, we will focus mainly on efficient staff education and sufficient management of the trial by the study team. All medical records (MRs) of included patients will be appropriately labelled with brightly coloured stickers indicating the study group, monitored or standard therapy accordingly. Once per week, one of the team members will audit MRs to schedule appropriate assessments and laboratory samples. Additionally, every week, he/she will provide the attending physician with a list of planned interventions and assessments. We will organise bi-monthly departmental meetings to follow any concerns related to the trial.

At discharge, parents of included infants will receive oral and written instructions on vit D administration and scheduled follow-up appointments. Parents will receive a reminder text message 1 day before the scheduled visit. Caregivers of included infants will be free to contact the principal investigator at any time.

#### Data monitoring

A data monitoring committee has not been established, because the intervention in the trial (vit D 200–1000 IU) does not differ from the standard of care accepted by several paediatric societies [11–13]. The profile of potential side effects is also known.

### Sample size calculations

The sample size was calculated on the basis of the main outcome, defined as the number of neonates with deficient or excess levels of 25(OH)D at 40 ± 2 weeks of PCA [16]. To meet acceptable recruitment rates and to obtain statistically significant results, we chose to detect a decrease of 25% in the number of patients with VDD with a power of 80% and an α value of 0.05; hence, 57 infants are needed in each study group. In order to account for 20% loss to follow-up, we aim to recruit a total of 138 infants for the study.

### Statistical analysis

Statistical analysis will be performed using Statistica 13.1 software (StatSoft, Tulsa, OK, USA). We plan to perform intention-to-treat analyses. Continuous data will be expressed as means with SDs or as medians with ranges, whereas categorical variables will be expressed as proportions. Normally distributed continuous variables will be analysed using Student’s *t* test, whereas the Wilcoxon rank-sum test will be used for skewed data. Categorical variables will be analysed using the chi-square test or Fisher’s exact test. Confidence intervals (95% CIs) will be calculated for RR, as well as risk differences for categorical variables, and mean differences with 95% CIs will be calculated for continuous variables.

## Discussion

The need for vit D supplementation in both term and preterm infants is widely acknowledged [11–13]. Despite multiple years of research and numerous publications, there is still a lack of consensus regarding *how much* vit D infants should receive and *how long* they should receive it. Because 80% of calcium and phosphorus placental transfer occurs between 24 and 40 weeks of gestation, preterm infants are especially prone to adverse effects of vit D insufficiency. A recent publication revealed that women receiving hormonal contraceptives prior to conception are at higher risk of decreased 25(OH)D levels, and this may affect our results [31]. However, both inadequate and excessive amounts of vit D may be unsafe and lead to serious health issues. The results of our study may shed new light on these concerns and contribute to optimising vit D supplementation.

### Trial status

**Fig. 2:**
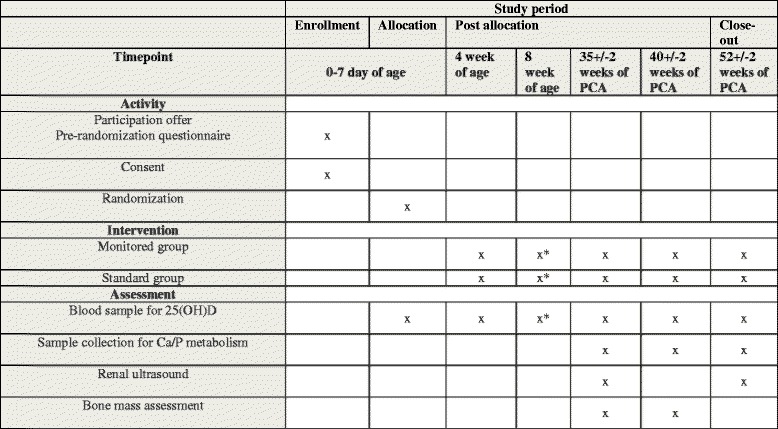
Schedule of enrolment, interventions and assessments. *25(OH)D* 25-Hydroxyvitamin D, *PCA* Post-conceptional age

Recruitment started in May 2017 and will last until May 2019. *Please see* Table 1 and Fig. 2 for all planned patient-related activities.

**Table 1:** Timetable of activities strictly related to participants planned throughout the study

Activity/assessment	0–7 DOL	4 WKA	8 WKA^a^	35 ± 2 weeks of PCA	40 ± 2 weeks of PCA	52 ± 2 weeks of PCA
Randomisation	+					
Blood sampling for 25(OH)D	+	+	+	+	+	+
Vit D dose adjustment		+	+	+	+	+
Sample collection for Ca^2+^/P metabolism				+	+	+
US kidney examination				+		+
Bone mass measurement				+	+	

*Abbreviations: DOL* Day of life, *Ca/P* Calcium/phosphate ratio, *25(OH)D* 25-Hydroxyvitamin D, *PCA* Post-conceptional age, *US* Ultrasound, *Vit D* Vitamin D, *WKA* Weeks of age

^a^Only infants born at < 26 weeks of gestational age

The manuscript, checklist and figures have been edited according to Standard Protocol Items: Recommendations for Interventional Trials (SPIRIT) guidelines [32] (Additional file 1).

## Additional file

Additional file 1: SPIRIT 2013 Checklist: Recommended items to address in a clinical trial protocol and related documents. (DOC 103 kb)

## Abbreviations

ABM: Average bone mass
ALP: Serum alkaline phosphatase
BPD: Bronchopulmonary dysplasia
CA: Corrected age
DOL: Days of life
ELBW: Extremely low birth weight
ESPGHAN: European Society for Paediatric Gastroenterology, Hepatology and Nutrition
GA: Gestational age
MBD: Metabolic bone disease
MR: Medical record
NEC: Necrotizing enterocolitis
25(OH)D: 25-Hydroxyvitamin D
P: Phosphate
PCA: Post-conceptional age
RR: Relative risk
SAE: Serious adverse event
SOS: Speed of sound
SPIRIT: Standard Protocol Items: Recommendations for Interventional Trials
TRP: Tubular reabsorption of phosphate
US: Ultrasound
VDD: Vitamin D deficiency
Vit D: Vitamin D
